# Discrepancies Between Autofluorescence Imaging Modalities in *CNGB3*-Associated Achromatopsia and Correlation With Ellipsoid Zone Continuity

**DOI:** 10.1167/tvst.14.5.24

**Published:** 2025-05-27

**Authors:** Haseeb N. Akhtar, Chun Fung Jeffrey Lam, Siying Lin, Gavin Arno, Jose S. Pulido, Andrew R. Webster, Michel Michaelides, Omar A. Mahroo

**Affiliations:** 1 UCL Institute of Ophthalmology, University College London, London, UK; 2 Genetics Service, Moorfields Eye Hospital, London, UK; 3 Section of Ophthalmology, King's College London, St Thomas’ Hospital Campus, London, UK; 4 Division of Evolution, Infection and Genomics, The University of Manchester, Manchester, UK; 5 Manchester Centre for Genomic Medicine, Saint Mary's Hospital, Manchester University NHS Foundation Trust, Manchester, UK; 6 Greenwood Genetic Center, Greenwood, SC, USA; 7 Department of Translational Ophthalmology, Wills Eye Hospital, Philadelphia, PA, USA; 8 Department of Physiology, Development and Neuroscience, University of Cambridge, Cambridge, UK

**Keywords:** retina, achromatopsia, inherited retinal disease

## Abstract

**Purpose:**

To explore discrepancies on fundus autofluorescence (FAF) obtained with two widely used devices in patients with *CNGB3*-associated achromatopsia, with respect to the central foveal signal. Secondly, to explore continuity of the foveal ellipsoid zone (EZ) in these patients.

**Methods:**

Patients who had undergone blue (488 nm; Heidelberg Spectralis) and green (532 nm; Optos) FAF imaging during the same visit were included. The central foveal signal was graded qualitatively as brighter or darker compared to the wider foveal/parafoveal region. Optical coherence tomography images from the same visit were also graded (masked to FAF grading) with respect to foveal EZ continuity.

**Results:**

Forty-one patients (24 females; mean age, 32 ± 19 years) were included. For blue FAF, the central foveal signal was graded darker in all cases. For green FAF, the central fovea was brighter in 11 patients (27%), indicating discordance with blue FAF. The discordant group were significantly younger (*P* = 0.022). The EZ line was gradable in 40 patients: 22 (55%) had continuous foveal EZ in both eyes; these were younger than those with an interrupted EZ in one or both eyes (*P* < 0.0001). All patients discordant for FAF images had continuous foveal EZ.

**Conclusions:**

Discordance occurred between the FAF modalities in more than one-quarter of patients; these patients were significantly younger, and all had a continuous EZ line. Investigating mechanisms of discordance could yield pathophysiological insights.

**Translational Relevance:**

FAF platforms are not interchangeable; these findings could inform the design of natural history studies and therapeutic trials for this condition.

## Introduction

Fundus autofluorescence (FAF) imaging frequently shows patterns of abnormality not evident on clinical examination or on other imaging modalities, and thus can be useful in diagnosis and monitoring of numerous retinopathies, including age-related macular degeneration, inherited retinal diseases, and drug toxicity.^1^^–^^15^ Patterns of FAF change over time can provide insights into disease mechanisms, as well as the natural history and efficacy of novel therapies.

**Figure 1.**
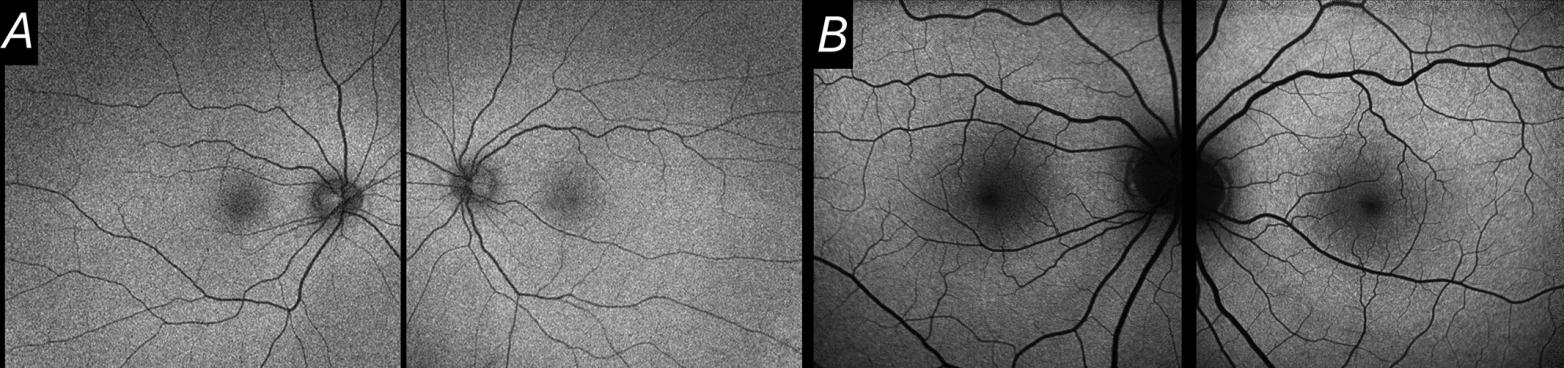
FAF images from a healthy individual (aged 25). (**A**) Green FAF images from both eyes. (**B**) Blue FAF images from both eyes. In both types of image, the central fovea is relatively hypoautofluorescent compared with the surrounding region.

Two imaging platforms widely used for FAF imaging are the Spectralis (Heidelberg Engineering, Heidelberg, Germany) and Optos (Optos, Dunfermline, UK) systems, which use exciting wavelengths of 488 nm and 532 nm, respectively.^3^ Figure 1 shows example images obtained from a healthy individual. These devices are used interchangeably sometimes, and in many diseases they give broadly similar qualitative or quantitative findings. However, differences exist between the two modalities. For example, in some patients with achromatopsia, we have observed a discrepancy whereby the central fovea can show relatively increased or decreased autofluorescence signal (compared with the parafoveal region) in the same patient depending on which imaging system is used. Here, we investigated this systematically in patients with *CNGB3*-associated achromatopsia. We also explored whether discrepancy between the two imaging modalities might correlate with ellipsoid zone (EZ) continuity on optical coherence tomography (OCT) imaging.

## Methods

A retrospective search was undertaken of the electronic patient record of the inherited retinal disease patient cohort at a single large centre (Moorfields Eye Hospital),^16^^,^^17^ to identify patients with achromatopsia. The gene most frequently involved was *CNGB3*. Patients with molecularly confirmed *CNGB3*-associated achromatopsia who had undergone both types of FAF imaging (blue [488 nm; Heidelberg Spectralis] and green [532 nm; Optos]) at the same visit were included in the study. If there were two or more visits where both modalities had been used, the most recent visit with gradable images was included. The central foveal signal was graded qualitatively in each eye as hyperautofluorescent or hypoautofluorescent relative to the wider foveal and parafoveal region. A specific aim was to identify eyes with discrepancy between the two modalities; these were labelled discordant.

Second, macular OCT images from the same visit were analyzed. Another qualitative binary classification was undertaken: for the OCT scan through the foveal centre in each eye, the EZ line was graded as continuous or discontinuous. This grading was done by a different investigator, masked to the results of the FAF classification. We explored whether patients exhibiting discordance on FAF images fell predominantly in either of the two OCT classifications.

This study adhered to the tenets of the Declaration of Helsinki. Patients gave written informed consent for genetic testing. Research ethics approval was from Moorfields Eye Hospital and the Northwest London Research Ethics Committee.

## Results

### Patient Demographics

Our search yielded 41 patients (24 females; 17 males) with molecularly confirmed *CNGB3*-associated achromatopsia who had undergone imaging with both FAF devices during the same clinic visit. The mean age was 32 ± 19 years (median, 25 years; range, 8–73 years).

### FAF Binary Grading and Concordance

All patients had the same qualitative grading for right and left eyes. For blue AF images, the central foveal signal was graded as darker (relatively hypoautofluorescent) compared with the remainder of the foveal/parafoveal area in all cases. For green AF images, the central foveal signal was darker in most, but brighter (relatively hyperautofluorescent) in 11 patients (27%; 4 females, 7 males), indicating discordance between the two platforms in this respect.

**Figure 2.**
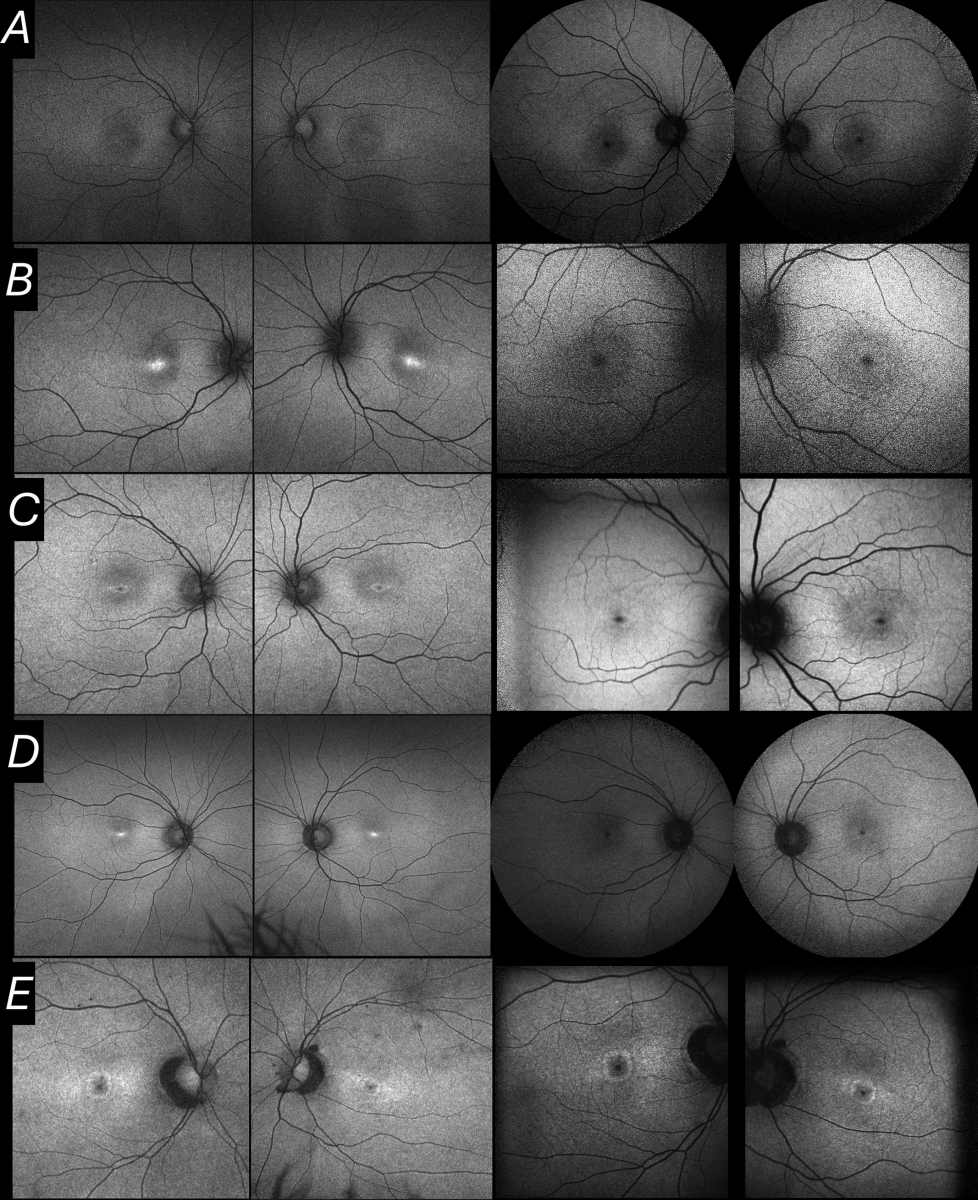
Examples of FAF images. In each row, images are from the same patient at the same visit. (*left*) Green FAF images. (Right) Blue FAF images. (Rows B and D were graded as discordant.) (**A**) A 13-year–old boy: central foveal signal graded as hypoautofluorescent for both modalities. (**B**) An 18-year-old man: central foveal signal graded as hyperautofluorescent for green FAF and hypoautofluorescent for blue FAF. (**C**) A 22-year-old woman: central foveal signal graded as hypoautofluorescent for both modalities. (**D**) A 25-year-old woman: central foveal signal graded as hyperautofluorescent for green FAF and hypoautofluorescent for blue FAF. (**E**) A 48-year-old woman: central foveal signal graded as hypoautofluorescent for both modalities.

**Figure 3.**
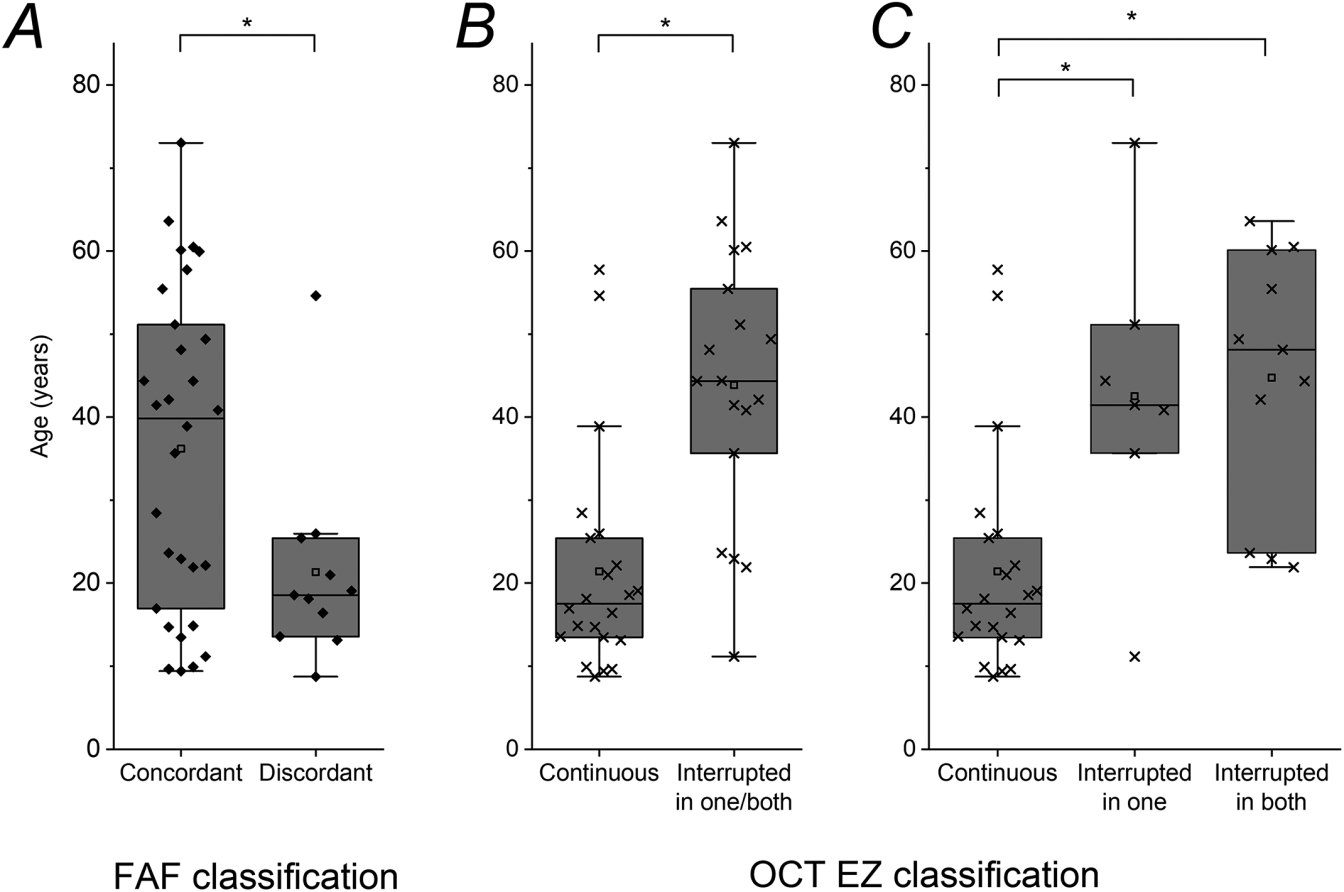
Box plots showing ages of patients by group. Boxes show median and interquartile range; whiskers show 1.5× interquartile range, with all data points (including outliers) also shown. Mean values are shown by open squares. *Significant differences. (**A**) FAF classification. Patients discordant between the two FAF modalities were significantly younger than those who were concordant (*P* = 0.022). (**B**) OCT grouping as to whether the foveal EZ was continuous in both eyes or interrupted in one or both eyes. Patients with continuous foveal EZ in both eyes were significantly younger than those with interrupted EZ in one or both eyes (*P* < 0.0001). (**C**) OCT grouping with subdivision of latter group into those in whom the EZ was interrupted in one eye only and those in whom the EZ was interrupted in both eyes. Patients with continuous foveal EZ in both eyes were significantly younger than those with interrupted EZ in one eye (*P* = 0.0026) and those with interrupted EZ in both eyes (*P* < 0.0001). The age difference between the latter two groups was not significant.

Figure 2 shows examples of concordant and discordant individuals. Figure 3A shows the age distributions of the two groups. The mean age of the concordant group was 36 ± 19 years (median, 39 years; range, 9–73 years). The mean age for the discordant group was 21 ± 12 years (median, 18 years; range, 8–54 years). The patients in the discordant group were significantly younger (*P* = 0.022).

### OCT EZ Line Binary Grading

The central EZ line was gradable in 40 patients (98%). This was graded as interrupted in 14 right eyes (35%) and 15 left eyes (37.5%), and continuous in the remainder. Thirty-three patients (83%) had the same grading for right and left eyes. Twenty-two patients (55%; 11 females, 11 males) were graded as having a continuous foveal EZ line in both eyes. Eighteen patients (45%; 12 females, 6 males) were graded with an interrupted EZ line in one or both eyes (for 7 patients, the interrupted EZ line was in just one eye). Figure 3B shows the ages of the two groups. The mean age for those graded with a continuous EZ line in both eyes was 21 ± 13 years (median, 17 years; range, 8–57 years). The mean age for the remainder was 43 ± 16 years (median, 44 years; range, 11–73 years). Those with an interrupted EZ line in one or both eyes were significantly older (*P* < 0.0001). Figure 3C subdivides the interrupted EZ group into those with interruption in one eye (mean age, 43 ± 18 years; median, 41 years) and those with interruption in both eyes (mean age, 45 ± 16 years; median, 48 years). Those with continuous EZ in both eyes were significantly younger than each of the other groups (*P* values given in Fig. 3 legend).

**Figure 4.**
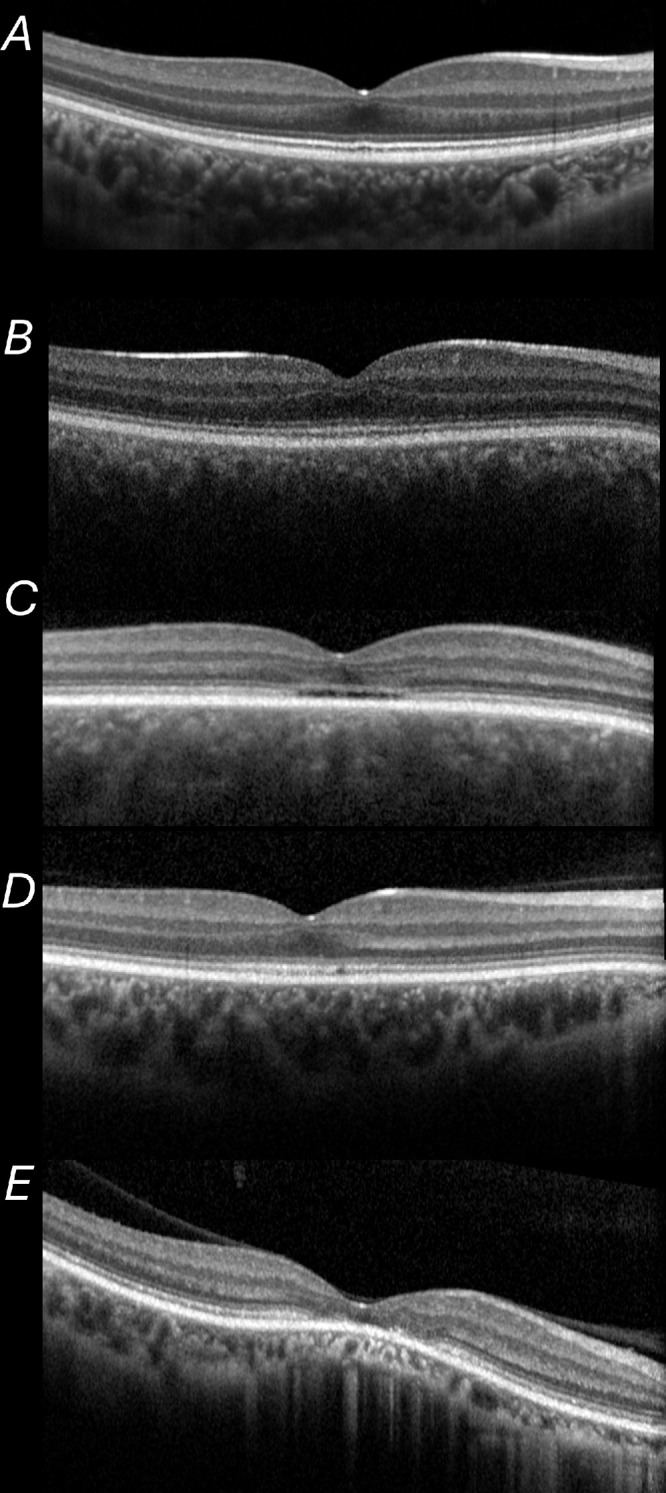
Examples of foveal OCT scans. Scans are from right eyes of the patients whose FAF images were depicted in Figure 1. The foveal EZ line was graded as continuous in (**A**, **B** and **D**), and as interrupted in (**C** and **E**).

Examples of OCTs are shown in Figure 4; these correspond with the patients whose FAF findings are depicted in Figure 2. All patients who were discordant for FAF grading were in the group in whom the EZ line was continuous centrally. Table gives numbers falling within the six possible groups defined with respect to both criteria (for the 40 patients for whom gradable FAF and OCT scans were available).

**Table.** Numbers of Patients Falling Into Groups Defined According to Both Criteria

	FAF Classification	
	Concordant (*n* = 29)	Discordant (*n* = 11)
Foveal EZ line on OCT		
Continuous in both eyes (*n* = 22)	11	11
Interrupted in one eyes (*n* = 7)	7	0
Interrupted in both eyes (*n* = 11)	11	0

Discordant FAF classification refers to those in whom the central foveal signal was relatively hyperautofluorescent (compared with the surrounding foveal/parafoveal area) on green FAF imaging, but relatively hypoautofluorescent on blue FAF imaging. Numbers are shown for a total of 40 patients. (One additional patient who was graded as concordant for FAF had poor quality OCTs precluding reliable EZ line grading.)

## Discussion

We explored discrepancies between FAF images acquired with two widely used devices, with respect to the central foveal signal, in patients with molecularly confirmed *CNGB3*-associated achromatopsia. In 27%, discordance was observed whereby the central fovea seemed to be relatively hyperfluorescent in green FAF images, but not in blue FAF images. These patients were significantly younger on average. No patients had an interocular discrepancy with respect to these grades. Second, we examined foveal OCT scans acquired at the same visit. In 45%, the EZ line was interrupted in one or both eyes; these patients were significantly older than those with continuous EZ lines in both eyes. The majority of the patients (83%) had the same OCT grading for both eyes. Interestingly, all patients found to be discordant for the central foveal signal on FAF (between the two devices) were within the group with continuous EZ lines in both eyes.

There have been several previous studies investigating FAF and OCT characteristics in achromatopsia, with useful classification systems proposed.^18^^–^^22^ The present study had the specific aim of investigating, systematically, a discrepancy we had observed in some patients in the qualitative appearance of the central foveal autofluorescence signal obtained using the two FAF devices. We, therefore, used a binary classification for each image. We also sought to explore a possible association with structural integrity on OCT. EZ line preservation is a commonly used metric for outer retinal diseases, and so we used another binary classification with respect to whether this line was continuous or interrupted through the fovea. Future studies, possibly in larger, pooled multicenter cohorts, could take a more granular approach.

**Figure 5.**
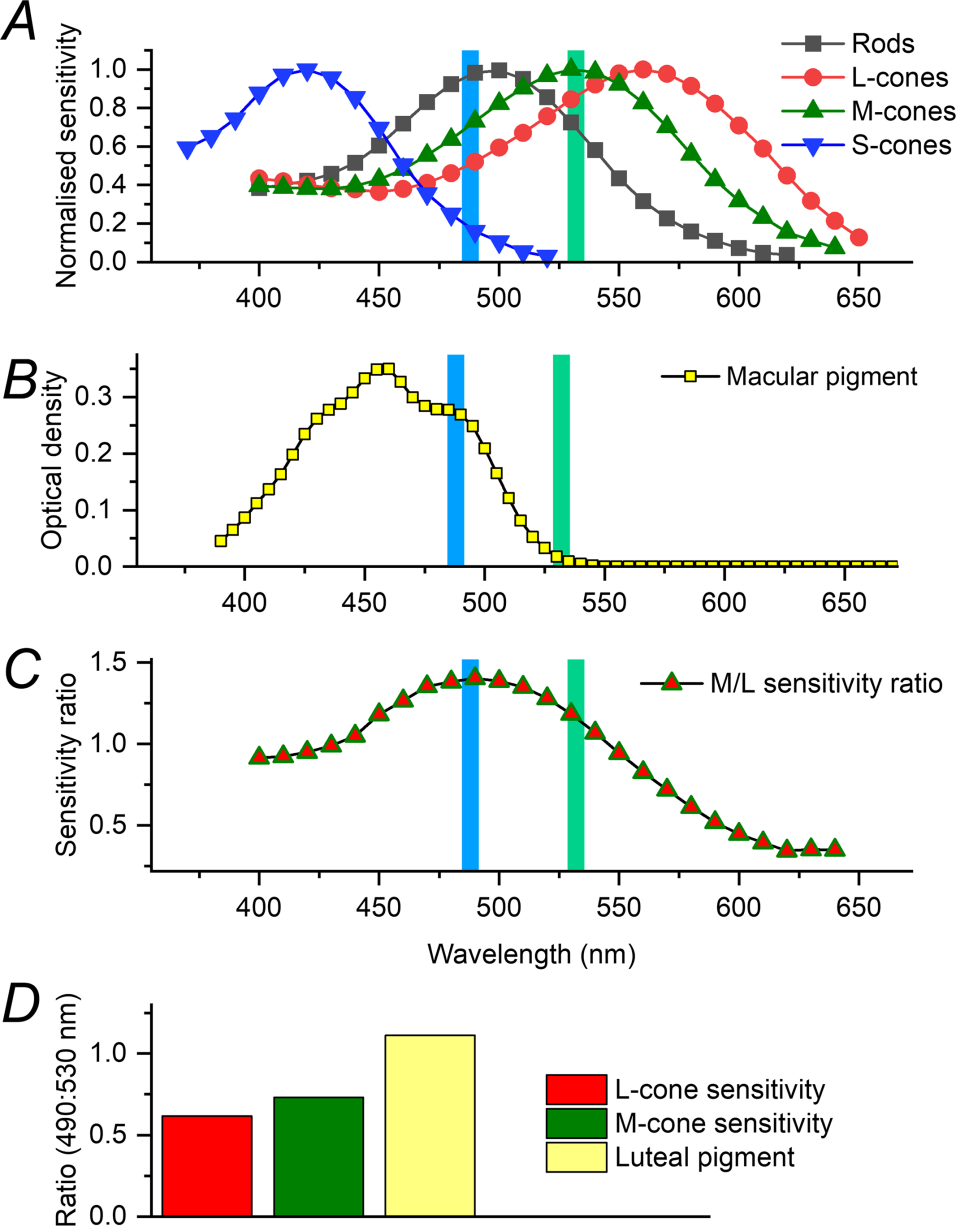
Spectral absorption data for photopigments and macular luteal pigment. In panels (**A**–**C**), the vertical *blue* and *green lines* show the blue and green FAF stimulating wavelength respectively. **(A**) Data are replotted from the study of Dartnall et al. of seven human eyes (their table 2), published in 1983.^23^ (**B**) Data are those of Bone et al.,^24^ as tabulated by Stockman et al. in 1999.^25^ These datasets are available at the Colour & Vision Research Laboratory website hosted at University College London (http://cvrl.ucl.ac.uk/ accessed 1 Jan 2025). (**C**) Ratio of normalized M-cone to L-cone sensitivity (taken from data points in A), plotted against wavelength. (**D**) Ratio (490:530 nm) for L-cone and M-cone sensitivities and luteal pigment optical density (calculated by dividing the corresponding value at 490 nm by that at 530 nm in **A** and **B**). These wavelengths were chosen as being the closest wavelengths to the FAF stimulation wavelengths of 488 and 532 nm for which data were available.

The precise mechanism underlying the discordance is uncertain. Given the difference in stimulation wavelengths, it is relevant to explore absorption spectra of macular luteal pigments and the photopigments. Figure 5 plots spectral sensitivity for the rod and cone photopigments (top)^23^ and optical density as a function of wavelength for macular luteal pigment (middle).^24^^,^^25^ Although actual absorption spectra differ between individuals and by technique, these data are likely to be broadly applicable.

The green stimulation wavelength (denoted by the vertical green line in both panels) would not be expected to be show significant absorption by luteal pigment, whereas absorption by L- and M-cone pigment (particularly M-opsin) would be substantial (also by rods, but these are absent from the fovea; S-cones are also absent from the central fovea). The shorter stimulation wavelength (denoted by the vertical blue line in both panels) would be expected to be absorbed substantially by macular pigment and may be absorbed also by L- and M-cone pigments, particularly M-opsin (but to a lesser extent than the green wavelength). Greater absorption by these pigments will diminish the amount of stimulating light reaching the retinal pigment epithelium fluorophores and hence will reduce the autofluorescence signal. One might hypothesize that those patients in whom discordance was observed have relatively diminished central foveal L- and M-opsins, but intact macular luteal pigment. It is possible that, later in life, as the foveal architecture is more disrupted, this difference no longer pertains, or overall fluorophore levels are lower in the retinal pigment epithelium (given that much of this is derived from photopigments) and so detectable discordance is less likely. This hypothesis would align with the younger age of patients in whom discordance was observed and the relative preservation of outer retinal integrity in this group.

Adaptive optics imaging studies have shown abnormalities of the cone mosaic in achromatopsia.^26^^–^^28^ Numbers of cone photoreceptors are reduced compared with age-matched controls, and the cones that are present appear to have altered reflectivity.^26^ There seems to be a marked degree of variability between patients, even with variants in the same gene.^27^ Such findings might lend support to the hypothesis that the variable findings between patients on green AF relate to variable degrees of loss or disruption of cone outer segment structure (including levels of L- and M-opsins). The central signal on blue AF (whose excitation wavelength would be less absorbed by L and M pigments) might be less prone to such variation.

Individuals also differ in their macular pigment distributions^29^^–^^31^ and in L:M-cone ratios;^32^^,^^33^ this might contribute to inter-individual differences. The third panel in Figure 5 plots the ratio of normalized M-to-L-cone sensitivity against wavelength. Although absorption at both wavelengths is greater for M opsin, the ratio at each wavelength is not identical: at 488 nm, normalized M-opsin sensitivity is approximately 40% greater than that for L-opsin, whereas at 532 nm, the corresponding figure is approximately 18%. Thus, an individual with relatively more M cones centrally might show different findings for the two FAF modalities from someone with a higher L:M-cone ratio. The bottom of Figure 5 shows ratios of absorption at 490 nm to 530 nm for L- and M-cones and for luteal pigment. This ratio is below 1 for L- and M-cones indicating that they will show greater absorption at 530 nm than at 490 nm (and this is more so for L-cones); for macular pigment, the ratio is greater than 1. Thus, differences between individuals in L:M ratios and macular pigment distributions can all contribute to the degree of discrepancy between green and blue AF relative intensities.

Our discussion has focused on differences in stimulation wavelength. The two devices also differ in other respects, including extent of signal averaging and degree of distortion of image dimensions (although the latter applies more to peripheral retina), and these could play a role. Also, the gain can be altered during image acquisition, so FAF patterns are necessarily relative. However, our study focused on comparing the central fovea with the immediate surrounding area; this comparison might be more robust to changes in overall gain. Future studies could potentially investigate further using quantitative autofluorescence techniques, for which an internal reference would be required.^34^ For example, relative central hypoautofluorescence can be both a feature of healthy individuals (Fig. 1) or advanced diseases where the retinal pigment epithelium is lost; more sensitive or quantitative classifications could potentially better identify these states. Newer models of the Optos system also allow blue FAF, and future studies comparing the two wavelengths using the same platform would be informative.

Discrepancies between the blue and green FAF modalities have been reported in the literature for healthy eyes and several pathologies, including geographic atrophy, *ABCA4* retinopathy, and various other retinochoroidopathies.^35^^–^^42^ In healthy eyes, more foveal hypoautoflourescence has been reported for blue compared with green FAF,^40^ and this difference is indeed evident in the healthy example shown in Figure 1. In geographic atrophy, some investigators have reported measured atrophic areas are higher with green FAF, though rates of progression may be similar.^41^ In the present study, we found an intriguing qualitative difference between the two types of autofluorescence in more than one-quarter of patients.

Our study focused on *CNGB3*-associated achromatopsia. *CNGB3* is the commonest gene associated with achromatopsia in our and several other cohorts.^16^^,^^17^^,^^43^^–^^45^ For the present analysis, we sought to constrain the number of variables by focusing on one particular genetic cause. However, we have anecdotally observed discrepancy in other conditions also, and so this finding should not be taken as specific to *CNGB3*-associated disease. The FAF discrepancies are likely to occur in patients with other forms of achromatopsia and likely in other conditions also, possibly with different degrees of correlation with EZ continuity; the pathophysiological basis for any discrepancies might also differ between diseases. Future studies can investigate this. Other limitations of our study include the cross-sectional rather than longitudinal nature. Future, larger studies could investigate change longitudinally (testing the hypothesis that discordance is a feature in earlier stages of disease) and potentially explore associations with different pathogenic variants.

A key implication of our study is that there can be qualitative differences between FAF platforms and hence devices should not be regarded as interchangeable. This is particularly important both for natural history studies and in the design of interventional trials. The findings also raise hypotheses regarding underlying mechanisms and it is possible that combining information from both modalities could yield insight into aspects of outer retinal physiology and pathophysiology in a range of retinal diseases.

## References

[1] von Rückmann A, Fitzke FW, Bird AC. Distribution of fundus autofluorescence with a scanning laser ophthalmoscope. *Br J Ophthalmol*. 1995; 79: 407–412, doi: 10.1136/bjo.79.5.407. 7612549 PMC505125

[2] Delori FC, Dorey CK, Staurenghi G, Arend O, Goger DG, Weiter JJ. In vivo fluorescence of the ocular fundus exhibits retinal pigment epithelium lipofuscin characteristics. *Invest Ophthalmol Vis Sci*. 1995; 36: 718–729. 7890502

[3] Yung M, Klufas MA, Sarraf D. Clinical applications of fundus autofluorescence in retinal disease. *Int J Retina Vitreous*. 2016; 2: 12. Published 2016 Apr 8, doi: 10.1186/s40942-016-0035-x. 27847630 PMC5088473

[4] Frampton GK, Kalita N, Payne L, et al. Fundus autofluorescence imaging: systematic review of test accuracy for the diagnosis and monitoring of retinal conditions. *Eye (Lond)*. 2017; 31: 995–1007, doi: 10.1038/eye.2017.19. 28282065 PMC5519265

[5] Heier JS, Lad EM, Holz FG, et al. Pegcetacoplan for the treatment of geographic atrophy secondary to age-related macular degeneration (OAKS and DERBY): two multicentre, randomised, double-masked, sham-controlled, phase 3 trials. *Lancet*. 2023; 402: 1434–1448, doi: 10.1016/S0140-6736(23)01520-9. 37865470

[6] Bassil FL, Colijn JM, Thiadens A, Biarnés M. Progression rate of macular retinal pigment epithelium atrophy in geographic atrophy and selected inherited retinal dystrophies. a systematic review and meta-analysis. *Am J Ophthalmol*. 2025; 269: 30–48, doi: 10.1016/j.ajo.2024.07.035. 39153684

[7] Heath Jeffery RC, Chen FK. Stargardt disease: multimodal imaging: a review. *Clin Exp Ophthalmol*. 2021; 49: 498–515, doi: 10.1111/ceo.13947. 34013643 PMC8366508

[8] Cicinelli MV, Rabiolo A, Brambati M, Viganò C, Bandello F, Battaglia Parodi M. Factors influencing retinal pigment epithelium-atrophy progression rate in Stargardt disease. *Transl Vis Sci Technol*. 2020; 9: 33. Published 2020 Jun 25, doi: 10.1167/tvst.9.7.33. PMC7414677 32832238

[9] Strauss RW, Ho A, Jha A, et al. Progression of Stargardt disease as determined by fundus autofluorescence over a 24-month period (ProgStar report no. 17). *Am J Ophthalmol*. 2023; 250: 157–170, doi: 10.1016/j.ajo.2023.02.003. 36764427

[10] Daich Varela M, Esener B, Hashem SA, Cabral de Guimaraes TA, Georgiou M, Michaelides M. Structural evaluation in inherited retinal diseases. *Br J Ophthalmol*. 2021; 105: 1623–1631, doi: 10.1136/bjophthalmol-2021-319228. 33980508 PMC8639906

[11] Corradetti G, Verma A, Tojjar J, et al. Retinal imaging findings in inherited retinal diseases. *J Clin Med*. 2024; 13: 2079. Published 2024 Apr 3, doi: 10.3390/jcm13072079. 38610844 PMC11012835

[12] Borrelli E, Bandello F, Boon CJF, et al. Mitochondrial retinopathies and optic neuropathies: the impact of retinal imaging on modern understanding of pathogenesis, diagnosis, and management. *Prog Retin Eye Res*. 2024; 101: 101264, doi: 10.1016/j.preteyeres.2024.101264. 38703886

[13] Lindeke-Myers A, Hanif AM, Jain N. Pentosan polysulfate maculopathy. *Surv Ophthalmol*. 2022; 67: 83–96, doi: 10.1016/j.survophthal.2021.05.005. 34000253

[14] Ahn SJ, Seo EJ, Kim KE, et al. Long-term progression of pericentral hydroxychloroquine retinopathy. *Ophthalmology*. 2021; 128: 889–898, doi: 10.1016/j.ophtha.2020.10.029. 33129843

[15] Lin S, Arno G, Robson AG, et al. Bifocal retinal degeneration observed on ultra-widefield autofluorescence in some cases of CRX-associated retinopathy. *Eye (Lond)*. 2025; 39: 951–957. Published online December 4, 2024, doi: 10.1038/s41433-024-03522-2. 39632990 PMC11933272

[16] Pontikos N, Arno G, Jurkute N, et al. Genetic basis of inherited retinal disease in a molecularly characterized cohort of more than 3000 families from the United Kingdom. *Ophthalmology*. 2020; 127: 1384–1394, doi: 10.1016/j.ophtha.2020.04.008. 32423767 PMC7520514

[17] Lin S, Vermeirsch S, Pontikos N, et al. Spectrum of genetic variants in the most common genes causing inherited retinal disease in a large molecularly characterized United Kingdom cohort. *Ophthalmol Retina*. 2024; 8: 699–709, doi: 10.1016/j.oret.2024.01.012. 38219857 PMC11932969

[18] Fahim AT, Khan NW, Zahid S, et al. Diagnostic fundus autofluorescence patterns in achromatopsia. *Am J Ophthalmol*. 2013; 156: 1211–1219.e2, doi: 10.1016/j.ajo.2013.06.033. 23972307

[19] Greenberg JP, Sherman J, Zweifel SA, et al. Spectral-domain optical coherence tomography staging and autofluorescence imaging in achromatopsia. *JAMA Ophthalmol*. 2014; 132: 437–445, doi: 10.1001/jamaophthalmol.2013.7987. 24504161 PMC4423754

[20] Sundaram V, Wilde C, Aboshiha J, et al. Retinal structure and function in achromatopsia: implications for gene therapy. *Ophthalmology*. 2014; 121: 234–245, doi: 10.1016/j.ophtha.2013.08.017. 24148654 PMC3895408

[21] Aboshiha J, Dubis AM, Cowing J, et al. A prospective longitudinal study of retinal structure and function in achromatopsia. *Invest Ophthalmol Vis Sci*. 2014; 55: 5733–5743. Published 2014 Aug 7, doi: 10.1167/iovs.14-14937. 25103266 PMC4161486

[22] Hirji N, Georgiou M, Kalitzeos A, et al. Longitudinal assessment of retinal structure in achromatopsia patients with long-term follow-up. *Invest Ophthalmol Vis Sci*. 2018; 59: 5735–5744, doi: 10.1167/iovs.18-25452. 30513534 PMC6280917

[23] Dartnall HJ, Bowmaker JK, Mollon JD. Human visual pigments: microspectrophotometric results from the eyes of seven persons. *Proc R Soc Lond B Biol Sci*. 1983; 220(1218): 115–130, doi: 10.1098/rspb.1983.0091. 6140680

[24] Bone RA, Landrum JT, Cains A. Optical density spectra of the macular pigment in vivo and in vitro. *Vision Res*. 1992; 32: 105–110, doi: 10.1016/0042-6989(92)90118-3. 1502795

[25] Stockman A, Sharpe LT, Fach C. The spectral sensitivity of the human short-wavelength sensitive cones derived from thresholds and color matches. *Vision Res*. 1999; 39: 2901–2927, doi: 10.1016/s0042-6989(98)00225-9. 10492818

[26] Dubis AM, Cooper RF, Aboshiha J, et al. Genotype-dependent variability in residual cone structure in achromatopsia: toward developing metrics for assessing cone health. *Invest Ophthalmol Vis Sci*. 2014; 55: 7303–7311. Published 2014 Oct 2, doi: 10.1167/iovs.14-14225. 25277229 PMC4235328

[27] Langlo CS, Patterson EJ, Higgins BP, et al. Residual foveal cone structure in CNGB3-associated achromatopsia. *Invest Ophthalmol Vis Sci*. 2016; 57: 3984–3995, doi: 10.1167/iovs.16-19313. 27479814 PMC4978151

[28] Katta M, Georgiou M, Singh N, et al. Longitudinal imaging of the foveal cone mosaic in CNGA3-associated achromatopsia. *Invest Ophthalmol Vis Sci*. 2024; 65: 6, doi: 10.1167/iovs.65.12.6. PMC11460564 39365261

[29] Berendschot TT, van Norren D. Macular pigment shows ringlike structures. *Invest Ophthalmol Vis Sci*. 2006; 47: 709–714, doi: 10.1167/iovs.05-0663. 16431971

[30] Delori FC, Goger DG, Keilhauer C, Salvetti P, Staurenghi G. Bimodal spatial distribution of macular pigment: evidence of a gender relationship. *J Opt Soc Am A Opt Image Sci Vis*. 2006; 23: 521–538, doi: 10.1364/josaa.23.000521. 16539047

[31] Tariq A, Mahroo OA, Williams KM, et al. The heritability of the ring-like distribution of macular pigment assessed in a twin study. *Invest Ophthalmol Vis Sci*. 2014; 55: 2214–2219. Published 2014 Apr 7, doi: 10.1167/iovs.13-13829. 24609627 PMC3979519

[32] Roorda A, Williams DR. The arrangement of the three cone classes in the living human eye. *Nature*. 1999; 397(6719): 520–522, doi: 10.1038/17383. 10028967

[33] Hofer H, Carroll J, Neitz J, Neitz M, Williams DR. Organization of the human trichromatic cone mosaic [published correction appears in J Neurosci. 2006 Jan 11;26:722]. *J Neurosci*. 2005; 25: 9669–9679, doi: 10.1523/JNEUROSCI.2414-05.2005. 16237171 PMC6725723

[34] Delori F, Greenberg JP, Woods RL, et al. Quantitative measurements of autofluorescence with the scanning laser ophthalmoscope. *Invest Ophthalmol Vis Sci*. 2011; 52: 9379–9390. Published 2011 Dec 9, doi: 10.1167/iovs.11-8319. 22016060 PMC3250263

[35] Wolf-Schnurrbusch UE, Wittwer VV, Ghanem R, et al. Blue-light versus green-light autofluorescence: lesion size of areas of geographic atrophy. *Invest Ophthalmol Vis Sci*. 2011; 52: 9497–9502. Published 2011 Dec 16, doi: 10.1167/iovs.11-8346. 22110076

[36] Nam KT, Yun CM, Kim JT, et al. Central serous chorioretinopathy fundus autofluorescence comparison with two different confocal scanning laser ophthalmoscopes. *Graefes Arch Clin Exp Ophthalmol*. 2015; 253: 2121–2127, doi: 10.1007/s00417-015-2958-6. 25690981

[37] Shin JY, Choi HJ, Lee J, Choi M, Chung B, Byeon SH. Fundus autofluorescence findings in central serous chorioretinopathy using two different confocal scanning laser ophthalmoscopes: correlation with functional and structural status. *Graefes Arch Clin Exp Ophthalmol*. 2016; 254: 1537–1544, doi: 10.1007/s00417-015-3244-3. 26690973

[38] Pfau M, Goerdt L, Schmitz-Valckenberg S, et al. Green-light autofluorescence versus combined blue-light autofluorescence and near-infrared reflectance imaging in geographic atrophy secondary to age-related macular degeneration [published correction appears in Invest Ophthalmol Vis Sci. 2018;59:674, doi: 10.1167/iovs.17-21764a]. *Invest Ophthalmol Vis Sci*. 2017; 58: BIO121–BIO130, doi: 10.1167/iovs.17-21764. 28632841

[39] Müller PL, Pfau M, Mauschitz MM, et al. Comparison of green versus blue fundus autofluorescence in ABCA4-related retinopathy. *Transl Vis Sci Technol*. 2018; 7: 13. Published 2018 Oct 1, doi: 10.1167/tvst.7.5.13. PMC6166893 30279998

[40] Bittencourt MG, Hassan M, Halim MS, et al. Blue light versus green light fundus autofluorescence in normal subjects and in patients with retinochoroidopathy secondary to retinal and uveitic diseases. *J Ophthalmic Inflamm Infect*. 2019; 9: 1. Published 2019 Jan 8, doi: 10.1186/s12348-018-0167-2. 30617430 PMC6325057

[41] Froines CP, Saunders TF, Heathcote JA, et al. Comparison of geographic atrophy measurements between blue-light Heidelberg standard field and green-light Optos Ultrawide Field Autofluorescence. *Transl Vis Sci Technol*. 2024; 13: 1, doi: 10.1167/tvst.13.11.1. PMC11540041 39495181

[42] Abbasgholizadeh R, Habibi A, Emamverdi M, et al. Comparison of Blue-light autofluorescence and ultrawidefield green-light autofluorescence for assessing geographic atrophy. *Ophthalmol Retina*. 2024; 8: 987–993, doi: 10.1016/j.oret.2024.04.017. 38670262

[43] Stone EM, Andorf JL, Whitmore SS, et al. Clinically focused molecular investigation of 1000 consecutive families with inherited retinal disease. *Ophthalmology*. 2017; 124: 1314–1331, doi: 10.1016/j.ophtha.2017.04.008. 28559085 PMC5565704

[44] Heutinck PAT, van den Born LI, Vermeer M, et al. Frequency and genetic spectrum of inherited retinal dystrophies in a large Dutch pediatric cohort: the RD5000 consortium [published correction appears in Invest Ophthalmol Vis Sci. 2024;65:26, doi: 10.1167/iovs.65.11.26]. *Invest Ophthalmol Vis Sci*. 2024; 65: 40, doi: 10.1167/iovs.65.10.40. PMC11361385 39189993

[45] Andersen MKG, Bertelsen M, Gundestrup S, Grønskov K, Kessel L. Phenotypic characteristics of Danish patients with achromatopsia. *Acta Ophthalmol*. 2024; 102: e893–e905, doi: 10.1111/aos.16656. 38348755

